# Body composition and risk of liver cancer: a population-based prospective cohort study on gender difference

**DOI:** 10.3389/fnut.2023.1102722

**Published:** 2023-05-15

**Authors:** Sainan Pi, Anran Liu, Beibei Zhu, Yunxiao Zhu, Jinqiu Yuan, Zheming Zhang, Chang Gao, Jinxian Fu, Yao Liu, Xujing Liang, Bin Xia, Youpeng Chen

**Affiliations:** ^1^Department of Infectious Diseases, The Seventh Affiliated Hospital, Sun Yat-sen University, Shenzhen, Guangdong, China; ^2^Department of Clinical Nutrition, The Seventh Affiliated Hospital, Sun Yat-sen University, Shenzhen, Guangdong, China; ^3^Endoscopy Center, The First Affiliated Hospital, Jinan University, Guangzhou, Guangdong, China; ^4^Health Management Center, The Seventh Affiliated Hospital, Sun Yat-sen University, Shenzhen, China; ^5^Clinical Research Center, The Seventh Affiliated Hospital, Sun Yat-sen University, Shenzhen, Guangdong, China; ^6^Big Data Center, The Seventh Affiliated Hospital, Sun Yat-sen University, Shenzhen, Guangdong, China; ^7^Center for Digestive Disease, The Seventh Affiliated Hospital, Sun Yat-sen University, Shenzhen, Guangdong, China; ^8^Department of Infectious Diseases, The First Affiliated Hospital, Jinan University, Guangzhou, Guangdong, China

**Keywords:** fat-free mass, cohort study, UK biobank, liver cancer, fat mass

## Abstract

**Background:**

Obesity is a common and highly convincing risk factor for many cancers, including liver cancer. Sex disparities in the body composition and regulatory mechanisms involved in energy homeostasis may contribute to the difference in the incidence of cancer. However, evidence on the gender-specific association between body composition and liver cancer incidence is limited. We performed this study to investigate the linear and non-linear associations of body composition with liver cancer risk by gender.

**Materials and methods:**

This prospective analysis included 4,75,659 participants free of cancer, based on the UK Biobank. We used Cox proportional hazard models to calculate the hazard ratios (HRs) and 95% confidence intervals (CIs) after adjusting for potential confounders. Restricted cubic spline was performed to investigate the potential non-linear associations.

**Results:**

During a median follow-up, 275 cases (174 male patients and 101 female patients) of liver cancer were identified. Male patients in the highest body fat percentage group are more likely to develop liver cancer (HR = 1.89, 95% CI: 1.17–3.03) compared with those in the lowest group. The one-unit increase of whole-body fat mass, arm fat mass, and trunk fat mass was associated with 1.03-, 1.14-, and 1.05-fold increased risk of liver cancer in male subjects, respectively. U-shaped associations of body composition with liver cancer risk were observed in the female subjects. Both high and low levels of whole-body fat-free mass, particularly in the arm and trunk, were associated with an increased risk of liver cancer.

**Conclusion:**

This study found a gender-specific association between body composition and liver cancer risk and provided evidence for individualized weight management for the prevention of liver cancer.

## 1. Introduction

Liver cancer is the sixth most frequently occurring cancer in the world and the second most common cause of cancer mortality. Data from the GLOBOCAN database showed that liver cancer was responsible for over 8,41,000 new cases and 78,200 deaths in 2020 (1). Many risk factors for liver cancer, including hepatitis B virus (HBV) or hepatitis C virus (HCV), alcohol, aflatoxin B1, and metabolic associated fatty liver disease (obesity, type II diabetes), have been identified (2).

The prevalence of obesity-related liver cancer has been increasing in recent years and has become the second biggest cause of liver cancer globally. A recent report by the World Cancer Research Fund (WCRF) showed that a high body mass index (BMI) is associated with a higher risk of liver cancer (3). One meta-analysis of 26 prospective studies indicated that excess body weight or obesity was associated with an increased risk of primary liver cancer (4).

Obesity, as a modifiable carcinogenic factor, has been demonstrated with remarkable gender disparities in the incidence and the cumulative risk of obesity-related liver cancer (5–9). A prospective study of more than 9,00,000 adults indicated that men with a BMI of 35 kg/m^2^ exhibited a dramatic 4.52-fold increase in relative risk of death from liver cancer, while a modest 1.68-fold increase was observed in women (10). A cohort study of 5.24 million adults in the UK has further confirmed the significant modulation of hepatocellular cancer (HCC) incidence by gender (11). Deregulated signaling of sex hormones is considered to be one of the drivers of sexual dimorphism.

Most previous studies have focused on the association between conventional measure indicators of obesity such as body mass index (BMI) and waist circumference (WC). However, conventional measure indicators tend to be conservative compared to others, and they may not adequately predict body composition and fat distribution. Evidence indicated that body fat percentage (BFP) and fat mass (FM)/fat-free mass (FFM) also had associations with liver cancer as well as obesity-related biomarkers. A cohort study of 4,37,393 participants found that BFP was associated with an increased risk of liver cancer (12). Due to its inadequate adjustment for important confounders, such as complications, and lack of analysis for fat distribution, further studies are still required.

The UK Biobank is a large population-based prospective study that includes more than 0.5 million individuals aged 37–73 years from the UK between 2006 and 2010. This dataset has collected a wide range of information on sociodemographic factors, lifestyle, and anthropometric measurements, as well as clinical diagnoses (13). Based on the UK Biobank, our study aimed to investigate the gender-specific relationship between body composition and the risk of liver cancer and further assess the potential non-linear associations.

## 2. Materials and methods

### 2.1. Study design and data collection

Data were collected from the UK Biobank (application number 51671, approved August 2019). The study protocol and information about data access are available online, and the details of the recruitment have been published elsewhere (11). For this analysis, participants were excluded if they had any cancer diagnoses (except for non-melanoma skin cancer ICD-10 C44) prior to baseline assessment or had missing data on the measure of body composition (*n* = 26,868). All the participants had follow-up from the date of the recruitment until the earliest date of liver cancer diagnosis, the date of death, and the date of loss to follow-up or end of follow-up for cancer incidence. Finally, a total of 4,75,659 participants were included in this analysis, and 275 cases were diagnosed with liver cancer (the flowchart of study selection is shown in Supplementary Figure S1). Information on cancer diagnoses in the UK Biobank is provided by the Health and Social Care Information Centre for participants in England and Wales and the NHS Central Register for participants in Scotland. The cancer registration codes were used from the International Statistical Classification of Diseases Tenth Modification (ICD-10). The UK Biobank was approved by the Northwest Multi-Center Research Ethics Committee (MREC), the Patient Information Advisory Group (PIAG), and the Community Health Index Advisory Group (CHIAG).

### 2.2. Anthropometry and body composition

At the baseline interview, trained personnel collected data on body composition and size using a standard protocol. The Tanita BC-418MA hepatocellular cancer (HCC) body composition analyzer (Tanita, Tokyo, Japan) was used to measure the FM (kg) and FFM (kg). Dual-energy X-ray absorptiometry (DXA) was also used to evaluate the body composition of 5,170 participants. Standing height was measured using the Seca 202 device (Seca, Hamburg, Germany). The Wessex non-stretchable sprung tape measure (Wessex, United Kingdom) was used to measure the waist/hip circumference, while the waist-to-hip ratio was calculated by dividing waist circumference (cm) by hip circumference (cm).

### 2.3. Data analysis

Baseline characteristics were compared between the first (lowest) and the fourth (highest) quartiles of both whole-body fat mass (WBFM) and whole-body fat-free mass (WBFFM). We used Cox regression models to estimate hazard ratios (HRs) and 95% confidence intervals (CIs) for the association of body composition and liver cancer risk.

We treated FM/FFM as a catalog or continuous variables to evaluate their relationship with the subsequent risk of liver cancer. To control potential confounding effects, we stratified the analyses by age in the basic Cox regression model and further adjusted for ethnicity (white/non-white), height, education (the highest qualification achieved), index of multiple deprivations, alcohol consumption (daily or almost daily, one or two times a week, one to three times a month, special occasions only, three or four times a week, and never or unknown), smoking status (never, current, and previous), physical activity, portions of fruit and vegetable intake, menopause status (even on hormone replacement therapy), family history of cancer, and previous comorbidities (diabetes/hepatitis/liver cirrhosis/hypertension diagnosis) in the multivariable-adjusted model (model 2). In addition, we used a restricted cubic spline with four knots at the 5th, 35th, 65th, and 95th percentiles to investigate possible non-linearity in each FM/FFM–liver cancer association, in which the median was set as the reference.

### 2.4. Sensitivity analysis

We performed two sensitivity analyses to test the robustness of the results. First, we excluded cancer diagnosed during the first 2 years of follow-up to minimize reverse causality. Second, we used the complete-case analysis to verify the influence of missing data. Data analysis was conducted using the R software (version 3.5.0, R Foundation for Statistical Computing, Vienna, Austria). Statistical significance was defined as a *p*-value of <0.05.

### 2.5. Patient and public involvement statement

There was no public involvement in the study; we used publicly available or privately held data for the analysis.

## 3. Results

### 3.1. Baseline characteristics

This study included 4,75,659 participants with a median follow-up of 6.6 years. Over this period, 275 incident liver cancer cases (174 male patients and 101 female patients) were recorded. As the whole-body fat mass and whole-body fat-free mass level quartiles increased, the participants tended to be less physically active and had a higher rate of hypertension and diabetes. The participants with lower WBFM or WBFFM tended to be current smokers. The distribution of the study population characteristics by quartiles of WBFM/WBFFM is presented in Table 1.

**Table 1:** Baseline characteristics stratified by gender.

	Whole-body fat mass(kg)		Whole-body fat-free mass(kg)	
Characteristics	Quartile 1	Quartile 4	Quartile 1	Quartile 4
Male				
No of participants	53,400	53,824	52,717	54,485
Mean (SD) age, y	55.2 (8.38)	57.3 (7.87)	58.5 (7.91)	54.4 (8.09)
White, %	93.1	95.1	89.8	96.2
IDM, mean (SD)	17.3 (14.0)	19.6 (15.1)	19.5 (15.2)	17.7 (14.1)
Current smokers, %	15.1	11.3	15.3	11.6
One or more times/week drinks, %	77	74.3	75	76.3
MET, mean(SD),h/week	54 (52.67)	39.5 (45.67)	48.33 (50.33)	44.67 (48.5)
Mean (SD) fruit and vegetable intake, portions per day	2.83 (2.81)	2.58 (2.46)	2.61 (2.70)	2.70 (2.54)
TG, mean(SD),mmol/L	1.52 (0.888)	2.34 (1.26)	1.76 (1.03)	2.22 (1.26)
CHOL, mean(SD),mmol/L	5.45 (1.04)	5.37 (1.19)	5.52 (1.14)	5.41 (1.12)
Hypertension, %	68.7	90.5	77.7	84.7
Diabetes, %	3.0	13.4	5.4	9.8
ALI, %	0.1	0.1	0.1	0.1
Liver cirrhosis, %	0.1	0.1	0.1	0.1
Female				
No of participants	62,702	63,147	62,295	63,854
Mean (SD) age, y	54.6 (8.19)	56.5 (7.81)	57.9 (7.67)	54.5 (8.02)
White, %	94.3	93.1	91.8	94.1
IDM, mean (SD)	15.8 (12.7)	20.0 (15.1)	17.2 (13.6)	18.7 (14.5)
Post menopause, %	62.5	72.3	78.7	61.8
Current smokers,%	10.2	8.3	9.5	9.0
One or more times/week drinks, %	68.1	62.2	62.9	56.4
MET, mean(SD),h/week	48.3 (44.17)	34.5 (37.5)	43.5 (42)	38.67 (40)
Mean (SD) fruit and vegetable intake, portions per day	3.44 (2.75)	3.08 (2.48)	3.31 (2.73)	3.21 (2.60)
TG, mean(SD),mmol/L	1.19 (0.613)	1.88 (0.954)	1.43 (0.761)	1.73 (0.960)
CHOL, mean(SD),mmol/L	5.73 (1.06)	5.83 (1.16)	6.00 (1.12)	5.71 (1.12)
Hypertension, %	51.6	80.4	64.6	72.1
Diabetes, %	1.4	8.0	2.2	7.1
ALI, %	0.1	0.1	0.1	0.1
Liver Cirrhosis, %	0.1	0.1	0.1	0.1
OCT, %	3.8	2.0	2.1	3.1
HRT, %	8.0	6.2	7.5	6.9

IDM, index of multiple deprivations; MET, metabolic equivalent for task; TG, triglyceride; ALI, acute liver injury; CHOL, cholesterol; OCT, optical coherence tomography; HRT, hormone replacement therapy; SD, standard deviation.

### 3.2. Body composition and incidence of liver cancer

The associations between body composition and liver cancer were likely to vary from sex, and the results are shown in Table 2. For male patients, WBFM was associated with an increased risk of liver cancer in male individuals. After adjustment for potential confounders, the liver cancer risk in the highest quartile increased 1.69 times greater (adjusted HR = 1.69, 95% CI: 0.99–2.88) compared with those in the lowest quartile. In addition, per 1 SD increase in WBFM was associated with a 3% increased risk of liver cancer. While in female participants, as the WBFFM increased, the risk of liver cancer decreased in the second quartile (adjusted HR: 0.37, 95% CI: 0.19–0.71) compared with that in the lowest quartile. While, in the third quartile (adjusted HR: 0.52, 95% CI: 0.28–0.99) and the highest quartile (adjusted HR: 0.63, 95% CI: 0.29–1.37), the risk was increased compared with that in the second quartile.

**Table 2:** Associations between whole-body fat-free mass/whole-body fat mass and risk of liver cancer.

	Male			Female		
	Hazard Ratio(95% CI)			Hazard Ratio(95% CI)		
	No of cases/ Person-years	Model 1^a^	Model 2^b^	No of cases/ Person-years	Model 1^a^	Model 2^b^
WBFM (kg)						
Quartile 1	29/349876	1 (ref)	1 (ref)	23/414586	1 (ref)	1 (ref)
Quartile 2	21/348536	0.66 (0.38–1.16)	0.62 (0.35–1.1)	23/413692	0.89 (0.5–1.59)	0.83 (0.46–1.5)
Quartile 3	44/347690	1.33 (0.83–2.13)	1.17 (0.71–1.92)	28/413349	1.04 (0.6–1.81)	0.88 (0.48–1.6)
Quartile 4	77/348968	2.3 (1.5–3.52)**	1.69 (0.99–2.88)**	22/413922	0.85 (0.47–1.52)	0.59 (0.27–1.29)
*p-value for trend*		<0.01	0.02		0.687	0.833
*Continuous per 1-unit increase*		1.04 (1.03–1.06)***	1.03 (1.01–1.05)**		1 (0.98–1.02)	1 (0.97–1.03)
WBFFM (kg)						
Quartile 1	41/342288	1 (ref)	1 (ref)	36/410665	1 (ref)	1 (ref)
Quartile 2	42/352408	1.1 (0.72–1.7)	1.19 (0.75–1.89)	14/416562	0.42 (0.22–0.77)**	0.37 (0.19–0.71)**
Quartile 3	36/348849	1.04 (0.66–1.63)	1.1 (0.65–1.86)	20/409782	0.64 (0.37–1.11)	0.52 (0.28–0.99)**
Quartile 4	52/355901	1.73 (1.14–2.62)*	1.56 (0.83–2.93)	26/419056	0.88 (0.53–1.47)	0.63 (0.29–1.37)
*p-value for trend*		0.044	0.938		0.544	0.87
*Continuous per 1-unit increase*		1.02 (1–1.04)*	1 (0.97–1.04)		1.01 (0.97–1.05)	1.01 (0.94–1.08)

*0.01 ≤ *p*-value < 0.05; **0.001 ≤ *p*-value < 0.01; ***p-value < 0.001.

CI, confidence interval; WBFFM, whole-body fat-free mass; WBFM, whole-body fat mass; ref, reference.

a Model 1: Adjusted for age.

b Model 2: Additionally adjusted for survival time, height, education, ethnicity, index of multiple deprivations, drinking status, smoking status, physical activity, fruit and vegetable intake, and complications (diabetes, hypertension, liver cirrhosis/failure, and hepatitis).

### 3.3. Distribution of body composition and liver cancer incidence

**Figure 1:**
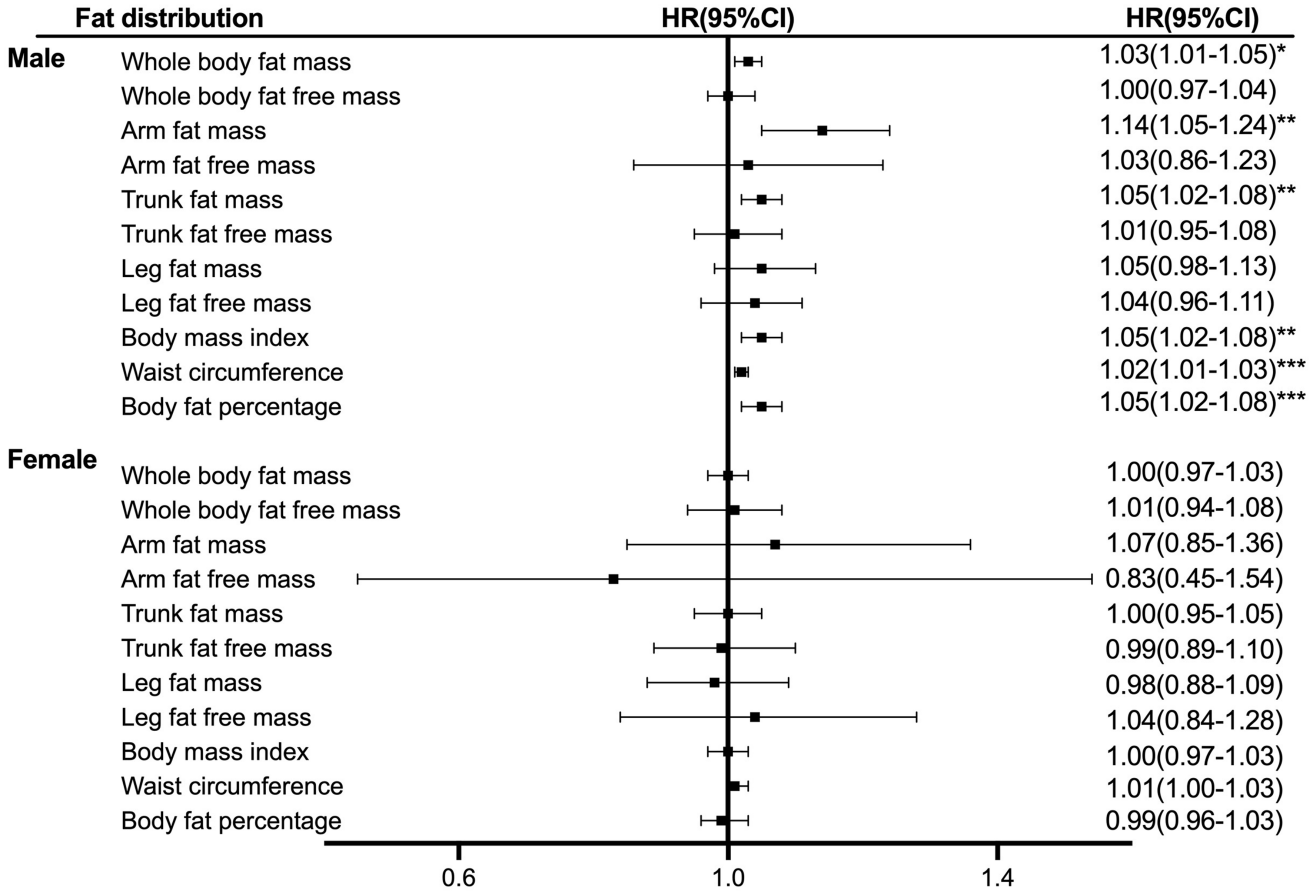
Hazard ratio per SD increase in liver cancer risk across anthropometric indices. The analyses were stratified age, survival time, education, ethnicity, index of multiple deprivations, drinking status, smoking status, physical activity, fruit and vegetable intake, and complications (diabetes, hypertension, liver cirrhosis/failure, and hepatitis).

The association between liver cancer risk and the distribution of body composition is presented in Figure 1. Arm fat mass in male patients showed a 14% per 1 SD increased risk of liver cancer (HR = 1.14, 95% CI, 1.05–1.24), followed by trunk fat mass (HR = 1.05, 95% CI, 1.02–1.08). There was no evidence of the linearity between body distribution with the risk of liver cancer for female patients.

### 3.4. Other anthropometric measures and liver cancer incidence

The risk of liver cancer was associated with BMI, WC, and BFP (Table 3). For male patients, a significant positive association with liver cancer incidence was observed per 1 SD increase in BMI (adjusted HR 1.05, 95% CI: 1.02–1.08), WC (adjusted HR 1.02, 95% CI:1.01–1.03), and BFP (adjusted HR 1.05, 95% CI: 1.02–1.08).

**Table 3:** Associations between BMI/WC and risk of liver cancer.

	Male			Female		
	Hazard Ratio(95% Confidence Interval)			Hazard Ratio(95% Confidence Interval)		
	No of cases/ Person-years	Model 1^a^	Model 2^b^	No of cases/ Person-years	Model 1^a^	Model 2^b^
BMI (kg/m^2^)						
Quartile 1	31/355454.6	1 (ref)	1 (ref)	25/420469.4	1 (ref)	1 (ref)
Quartile 2	23/355585.4	0.71 (0.41–1.21)	0.69 (0.40–1.18)	22/419704.3	0.8 (0.45–1.41)	0.78 (0.44–1.39)
Quartile 3	49/354715.7	1.50 (0.95–2.35)	1.35 (0.86–2.14)	25/419403.9	0.86 (0.49–1.5)	0.79 (0.45–1.39)
Quartile 4	71/354059.3	2.21 (1.45–3.36)***	1.72 (1.10–2.66)**	29/418469.6	1.03 (0.6–1.76)	0.87 (0.49–1.53)
*p-value for trend*		<0.01	0.02		0.072	0.26
*Continuous per 1-unit increase*		1.08 (1.05–1.11)***	1.05 (1.02–1.08)**		1.03 (1.00–1.07)	1.00 (0.97–1.03)
WC (cm)						
Quartile 1	27/322245	1 (ref)	1 (ref)	21/372914	1 (ref)	1 (ref)
Quartile 2	22/369011	0.65 (0.37–1.14)	0.61 (0.35–1.07)	27/446328	0.96 (0.54–1.7)	0.94 (0.53–1.67)
Quartile 3	40/343414	1.19 (0.73–1.94)	1.03 (0.63–1.69)	17/421672	0.6 (0.31–1.13)	0.55 (0.29–1.06)
Quartile 4	86/388642	2.2 (1.42–3.39)***	1.64 (1.05–2.58)**	36/439286	1.2 (0.7–2.07)	1.03 (0.58–1.81)
*p-value for trend*		<0.01	<0.01		0.04	0.16
*Continuous per 1-unit increase*		1.03 (1.02–1.05)***	1.02 (1.01–1.03)***		1.02 (1–1.03)	1.01 (1–1.03)
BFP (%)						
Quartile 1	24/344531	1 (ref)	1 (ref)	24/412805	1 (ref)	1 (ref)
Quartile 2	22/357146	0.79 (0.44–1.41)	0.74 (0.41–1.32)	19/416350	0.67 (0.37–1.23)	0.67 (0.36–1.22)
Quartile 3	41/346987	1.39 (0.84–2.31)	1.19 (0.71–1.99)	25/413535	0.84 (0.48–1.47)	0.81 (0.45–1.43)
Quartile 4	84/349324	2.6 (1.65–4.11)***	1.89 (1.17–3.03)**	28/413374	0.93 (0.54–1.62)	0.85 (0.47–1.51)
*p-value for trend*		<0.01	<0.01		0.544	0.87
*Continuous per 1-unit increase*		1.08 (1.05–1.11) ***	1.05 (1.02–1.08) ***		1 (0.97–1.03)	0.99 (0.96–1.03)

*0.01 ≤ *p*-value <0.05; **0.001 ≤ *p*-value <0.01; ***p-value <0.001.

CI, confidence interval; BMI, body mass index; BFP, body fat percentage; WC, waist circumference. Ref., reference.

a Model 1: Adjusted for age.

b Model 2: Additionally adjusted for survival time, height, education, ethnicity, index of multiple deprivations, drinking status, smoking status, physical activity, fruit and vegetable intake, and complications (diabetes, hypertension, liver cirrhosis/failure, and hepatitis).

### 3.5. Non-linear relationship between measures and the risk of liver cancer

**Figure 2:**
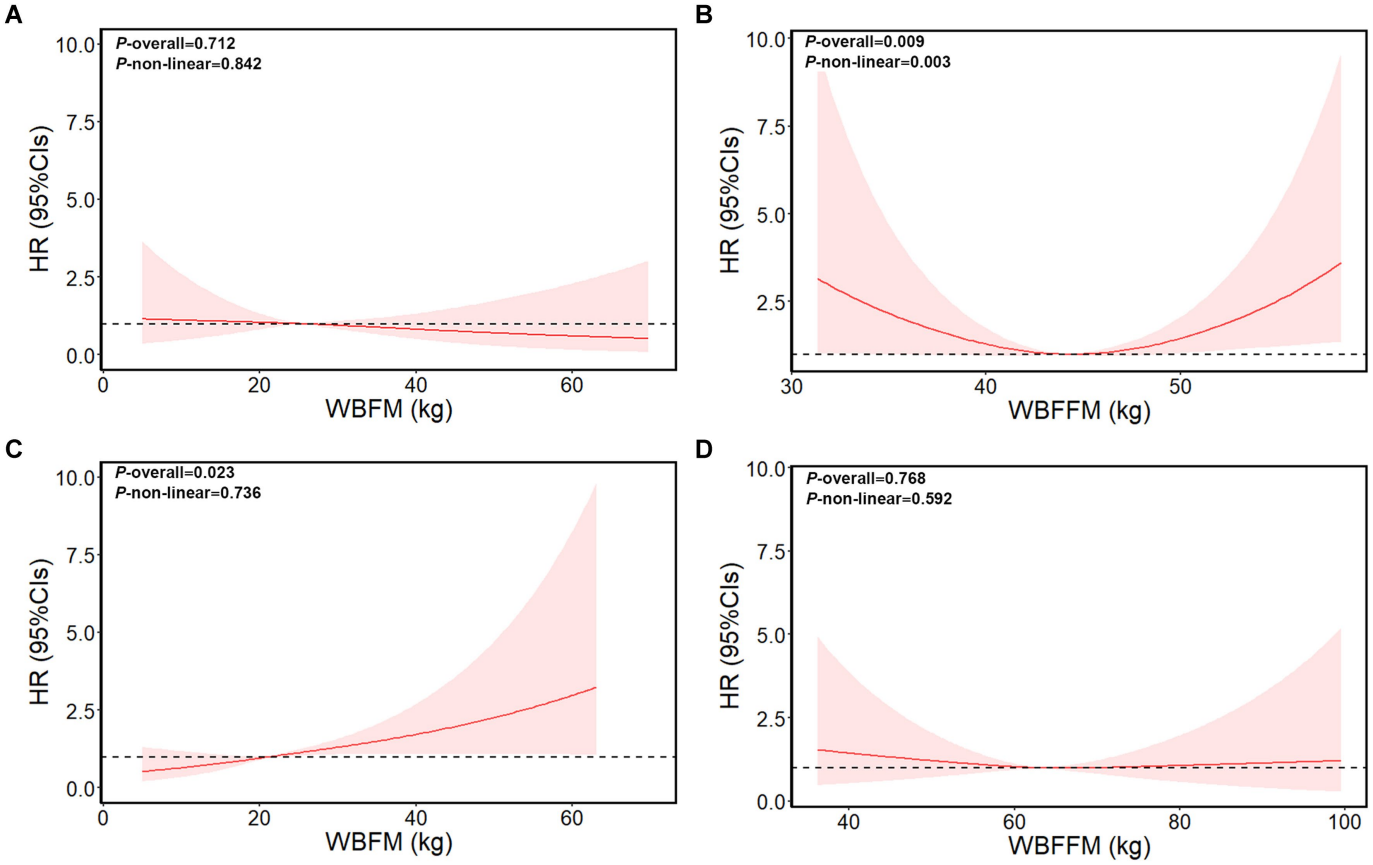
Dose–response relationship of whole-body fat mass/whole-body fat-free mass with liver cancer risk in female patients and male patients. **(A)** Whole-body fat mass of female patients. **(B)** Whole-body fat-free mass of female patients. **(C)** Whole-body fat mass of male patients. **(D)** Whole-body fat-free mass of male patients. The model adjusted for age, survival time, height, education, ethnicity, index of multiple deprivations, drinking status, smoking status, physical activity, fruit and vegetable intake, and complications (diabetes, hypertension, liver cirrhosis/failure, and hepatitis). WBFM: whole-body fat mass, WBFFM: whole-body fat-free mass.

We further evaluated the non-linear relationship between anthropometric indices markers and the risk of liver cancer (Figure 2; Supplementary Figures S2–S4). The liver cancer risk showed a U-shape relation with the markers including BMI, WC, WBFFM, arm fat-free mass, and trunk fat-free mass levels in female patients (*p*-overall <0.01, *p*-non-linear <0.01). Both higher and lower of these markers were associated with an increased risk of liver cancer. The nadir for incidence of liver cancer risk was estimated to be at a BMI of 27.3 kg/m^2^, a WC of 83 cm, a whole-body fat-free mass of 44 kg, an arm fat-free mass of 4.5 kg, and a trunk fat-free mass of 24.8 kg.

### 3.6. Sensitivity analyses

Appendix Supplementry Figure S5 shows the sensitivity analyses. By lagging the exposure for a time window of 2 years, the positive association between FM/FFM was stable. The result was unchanged even using the complete-case analysis to verify the influence of missing data.

## 4. Discussion

Based on this prospective cohort study of nearly half a million participants, we observed that BMI, WC, BFP, and FM distributed in the arm and the trunk were associated with an increased risk of liver cancer in male patients. However, in female participants, BMI, WC, and FFM distributed in the arm and the trunk had a U-shape relationship with liver cancer incidence. Collectively, these findings indicated a sexually dimorphic association between conventional measure indicators and body composition markers with liver cancer risk.

Prior studies showed that obesity contributes to the increased risk of liver cancer (3, 10, 14, 15). One meta-analysis, including 28 prospective cohort studies, reported that the incidence of liver cancer increased by approximately 36 and 77% in overweight adults and obese adults, respectively (16). A cohort study conducted in the UK found that 10% or more of liver cancer could be attributable to excess weight (7). Consistent with these studies, our data suggested a positive association of FM, as well as the BFP with increased risk of liver cancer in male patients, and also found the same effect with BMI and WC. However, we did not observe a linear relationship in female patients.

Body composition has shown sex differences. Women exhibit a higher tendency of deposition of fat in the form of subcutaneous adipose tissue (SAT), whereas in men, more fat tends to be deposited in the form of visceral adipose tissue (VAT) (17). VAT induces the production of not only circulating concentrations of insulin, free fatty acids (FFAs), and TG but also proinflammatory adipokines, such as leptin, tumor necrosis factor α (TNF-α), interleukin (IL)-6, and hypoxia-inducible factor, and immune cell infiltration, while SAT shows lower lipolysis activity, thus posing a lower risk of metabolic complications (18). Androgen promotes the development of liver cancer, while it should be noted that the signal transduction of estrogen and estrogen receptors might play a protective role in the initiation and progression of liver cancer (19). Estrogen has been proven to exert protective effects against HCC in the regulation of the inflammation network by restraining proinflammatory cytokines and inhibiting downstream signaling pathways.

There were few prospective studies on FFM and liver cancer risk. Epidemiological studies showed that FFM had related to the risk of malignancies, such as gastric and esophageal adenocarcinoma (20), rectal cancer (21), prostate cancer (22), and lung cancer (23). It has been believed that a greater FFM, and therefore, a greater resting metabolic rate, will protect against obesity and associated comorbidities (24). However, our study indicated that FFM, particularly in the arm and the trunk fat-free mass, had a U-shape association with cancer risks in female patients. FFM, mainly included skeletal muscle, is the main site of insulin consumption, and low muscle mass may contribute to the development of insulin resistance (IR) (25). As the muscle’s capacity to uptake the postprandial glucose is decreased, the glucose diverts from the muscles to the liver, leading to fat accumulation, which may increase the risk of carcinogenesis (26). The skeletal muscle mass is not only associated with the histological grades of steatosis and hepatocellular ballooning but also the stage of fibrosis. Patients with low muscle mass have approximately two times increased odds ratio of suffering from NASH or liver fibrosis. FFM depletion is an independent prognostic factor of liver cancer (27).

It is well known that the excess adipose tissue may result in insulin resistance, while the excess muscle also affected the sensitivity of insulin. Brochu et al. (28) showed that FFM was independently associated with changes in glucose uptake in obese female patients (29). Higher FFM demonstrated more severely impaired endothelial function and higher systemic inflammation as compared to the lower FFM (30). Further research studies are needed to test this novel concept.

As far as we know, there was a seldom report about the association between body composition and liver cancer in previous epidemiological studies. The main strength of our research is based on the large sample size prospective cohort with validated follow-up duration and detailed measurements. This allowed for simultaneous adjustability of potential confounders for the association of interest. In addition, we also investigated the potential non-linear relationship, which provided insight into the carcinogenicity of body composition and contributes to individualized cancer prevention.

This study had some limitations. First, participants in the UK Biobank were predominantly white individuals; therefore, the results of our study may not be generalizable to other ethnicities. Second, as an observational study, we may not exclude residual confounding effects completely and confirm the causal relationship. Third, due to a lack of histological information, the association of body composition with each subtype of liver cancer is not clear.

## 5. Conclusion

The available data suggested that body composition particularly in the arm and trunk tended to associate with an increased risk of liver cancer. Intentional weight loss may reduce the incidence of liver cancer in men, while limiting excessive fat-free mass gain may have benefits in reducing liver cancer in women. Our findings provided evidence for individualized weight management for the prevention of liver cancer. New body composition models and techniques for grading or predicting aspects of body composition are expected to be employed increasingly in future epidemiologic investigations of chronic disease morbidity and mortality. Further research is warranted to confirm our findings and to investigate the underlying mechanism of the gender-specified effects of FFM/FM on liver cancer development.

## Data availability statement

The raw data supporting the conclusions of this article will be made available by the authors, without undue reservation.

## Ethics statement

Written informed consent was obtained from the individual(s) for the publication of any potentially identifiable images or data included in this article.

## Author contributions

BX and YC designed the study, had full access to all study data, and conducted the research. YZ, JY, ZZ, CG, JF, YL, and XL performed data collection and statistical analyses. SP, AL, and BZ analyzed the data and wrote the manuscript. All authors contributed to the article and approved the submitted version.

## Funding

This study was supported by the National Natural Science Foundation of China (grant number 82003408 and 82003524) and the Startup Fund for the 100 Top Talents Program, SYSU (392012).

## Conflict of interest

The authors declare that the research was conducted in the absence of any commercial or financial relationships that could be construed as a potential conflict of interest.

## Publisher’s note

All claims expressed in this article are solely those of the authors and do not necessarily represent those of their affiliated organizations, or those of the publisher, the editors and the reviewers. Any product that may be evaluated in this article, or claim that may be made by its manufacturer, is not guaranteed or endorsed by the publisher.

## Glossary

ALI: acute liver injury
BFP: body fat percentage
BMI: body mass index
CHIAG: community Health Index Advisory Group
DXA: Dual-energy X-ray absorptiometry
HBV: hepatitis B virus
HCV: hepatitis C virus
HRs: hazard ratios
HRT: hormone replacement therapy
IL-6: interleukin-6
IDM: index of multiple deprivations
IR: insulin resistance
MREC: multi-center Research Ethics Committee
MET: metabolic equivalent for task
OCT: optical coherence tomography
PIAG: patient Information Advisory Group
TG: triglyceride
WC: waist circumference
WBFM: whole-body fat mass
WBFFM: whole-body fat-free mass
