# Supplementary figures and images for: Body composition and risk of liver cancer: a population-based prospective cohort study on gender difference
Source: Front Nutr. 2023 May 15;10:1102722. doi: 10.3389/fnut.2023.1102722 (PMC10234331; doi:10.3389/fnut.2023.1102722)

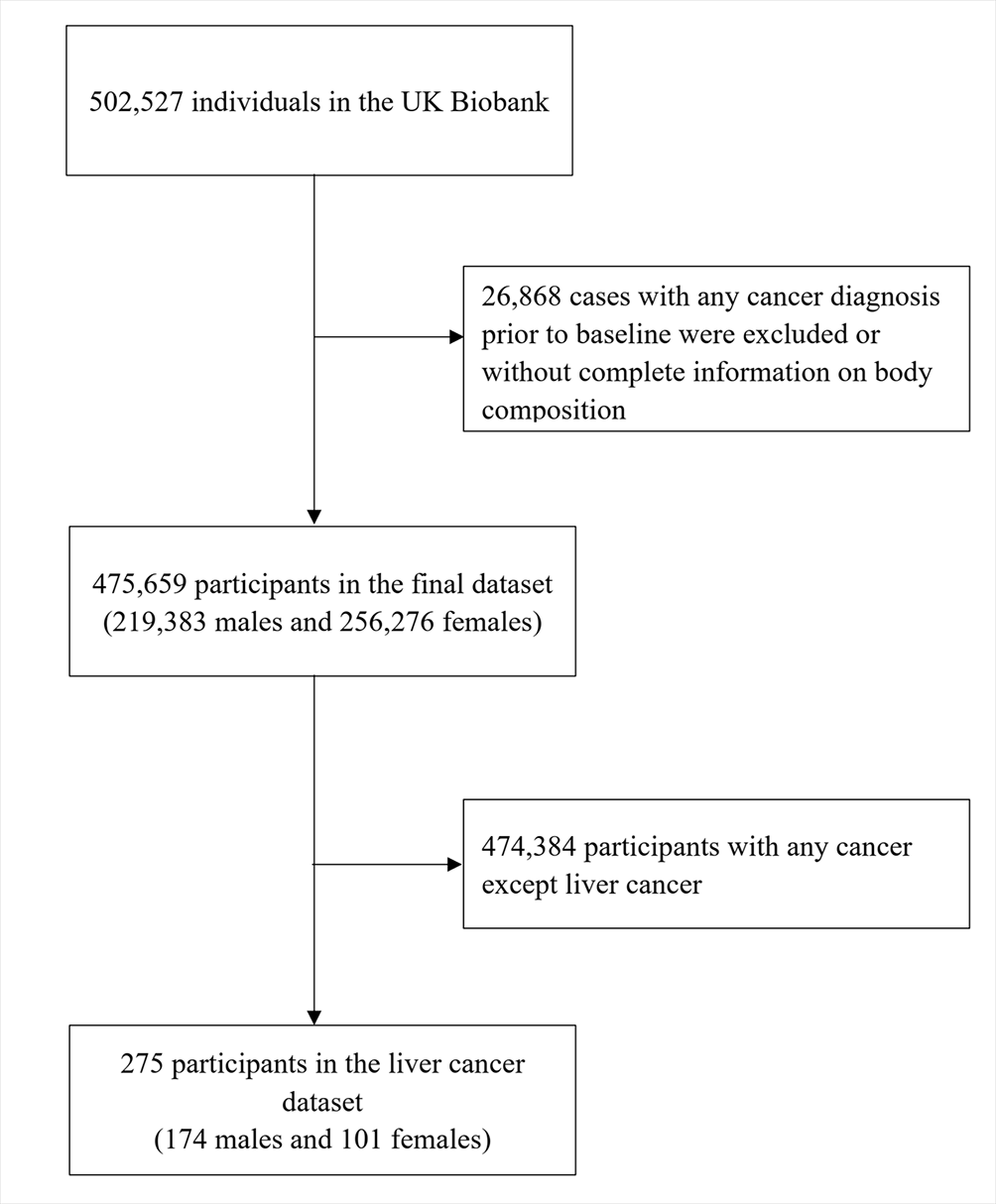

Supplement: Supplementary file 1 [file Data_Sheet_1.ZIP › Supplementary Figures/Supplementary Figure 1.tif]

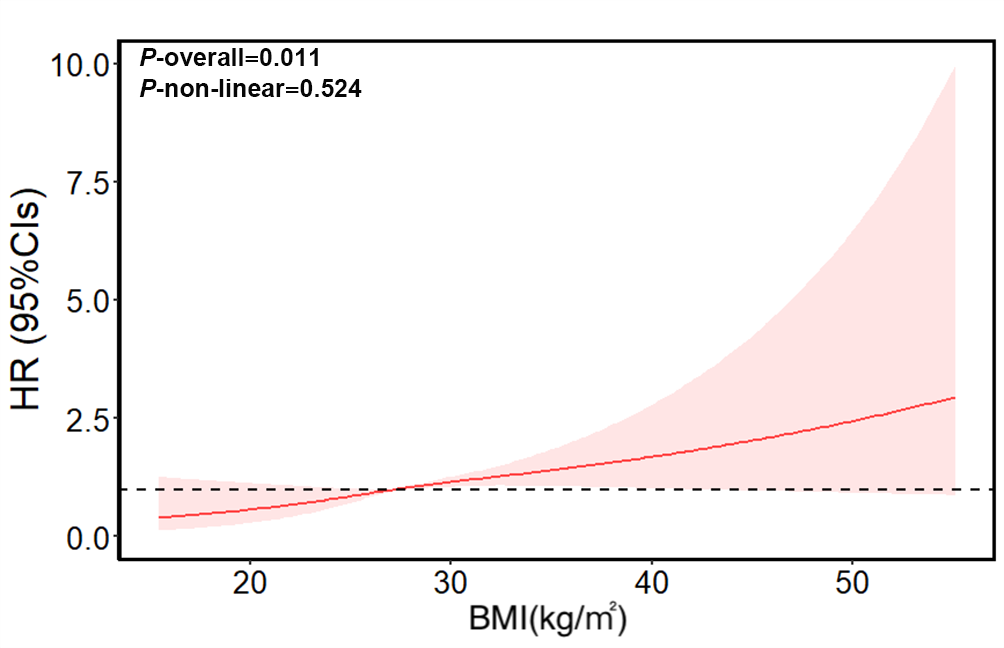

Supplement: Supplementary file 1 [file Data_Sheet_1.ZIP › Supplementary Figures/Supplementary Figure 2(A).tif]

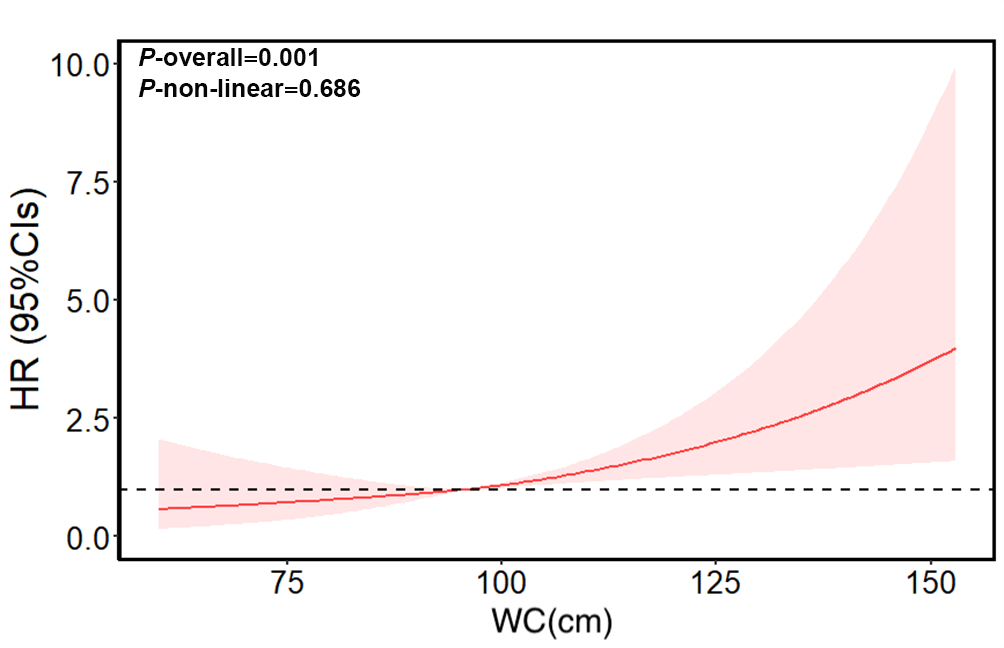

Supplement: Supplementary file 1 [file Data_Sheet_1.ZIP › Supplementary Figures/Supplementary Figure 2(B).tif]

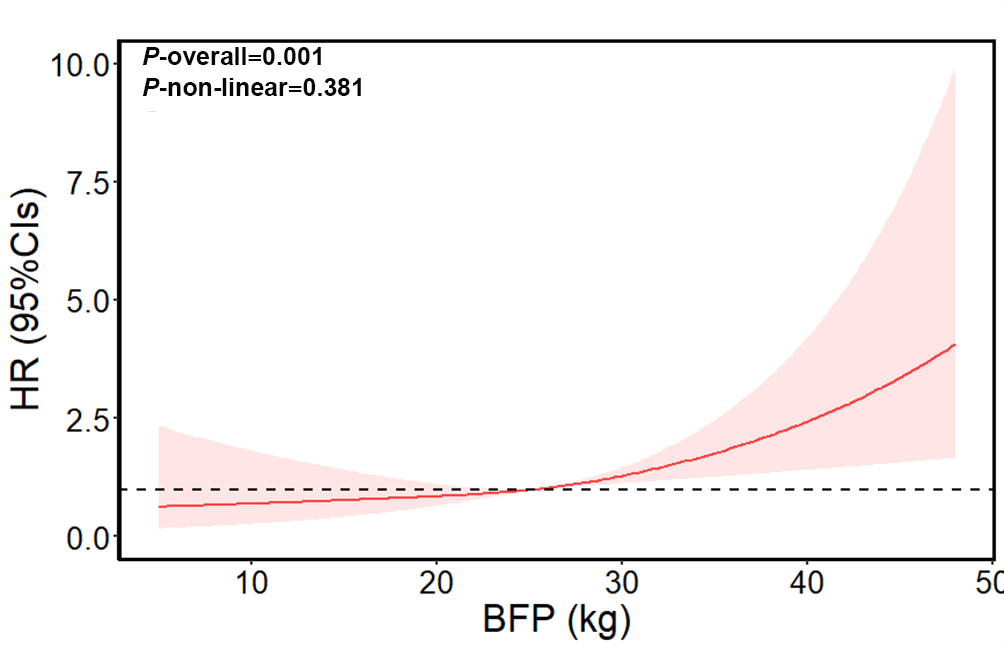

Supplement: Supplementary file 1 [file Data_Sheet_1.ZIP › Supplementary Figures/Supplementary Figure 2(C).tif]

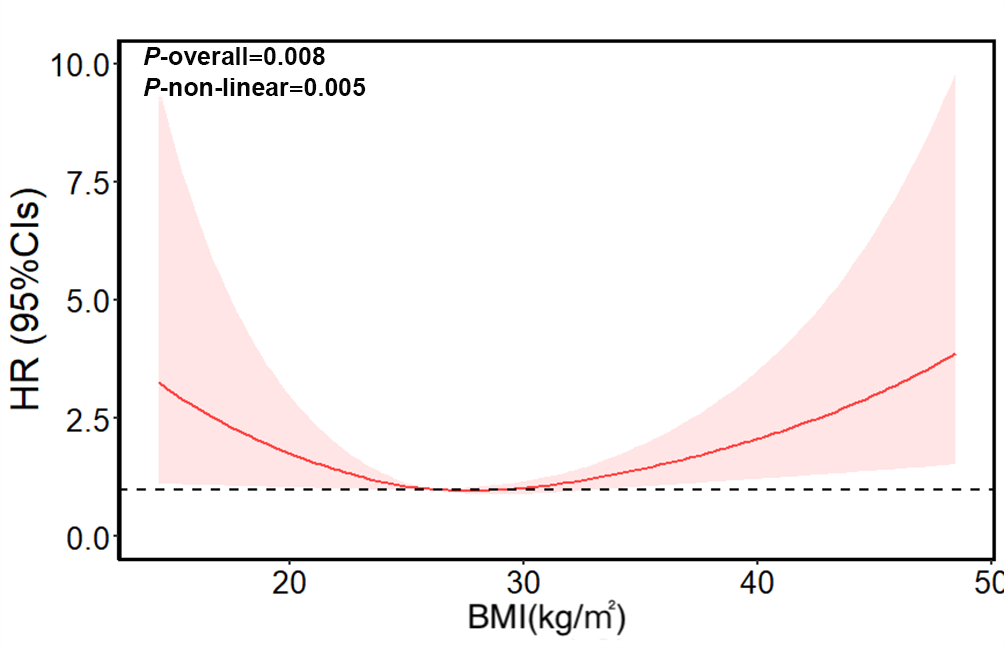

Supplement: Supplementary file 1 [file Data_Sheet_1.ZIP › Supplementary Figures/Supplementary Figure 2(D).tif]

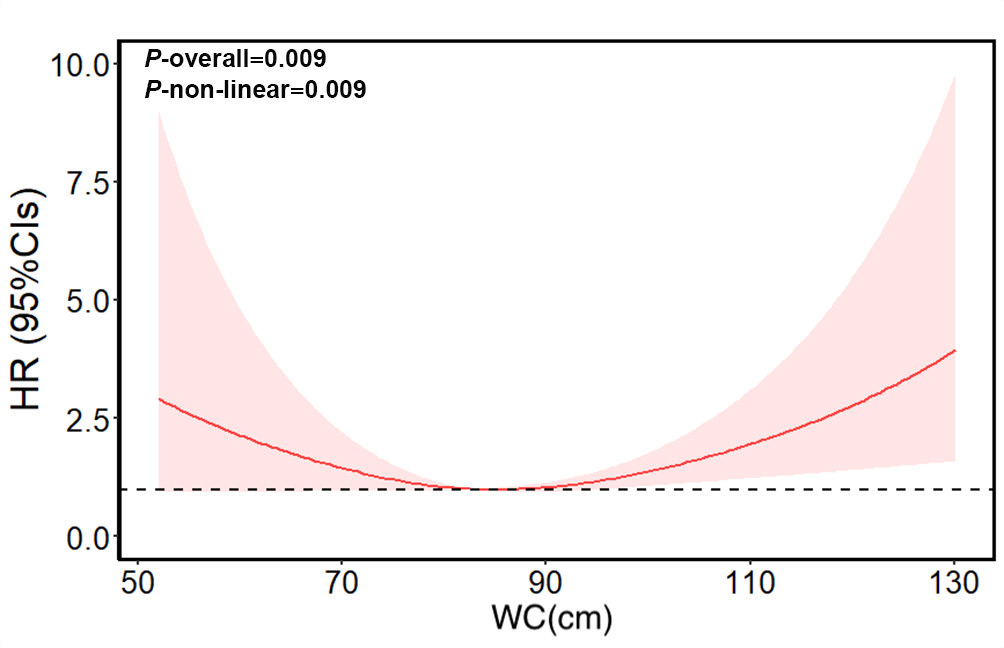

Supplement: Supplementary file 1 [file Data_Sheet_1.ZIP › Supplementary Figures/Supplementary Figure 2(E).tif]

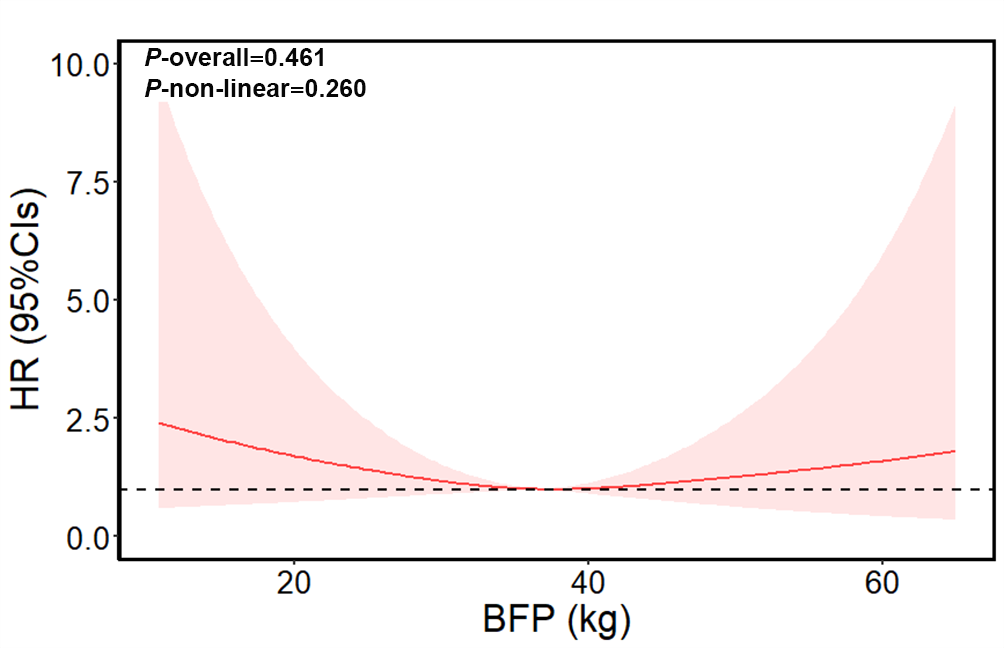

Supplement: Supplementary file 1 [file Data_Sheet_1.ZIP › Supplementary Figures/Supplementary Figure 2(F).tif]

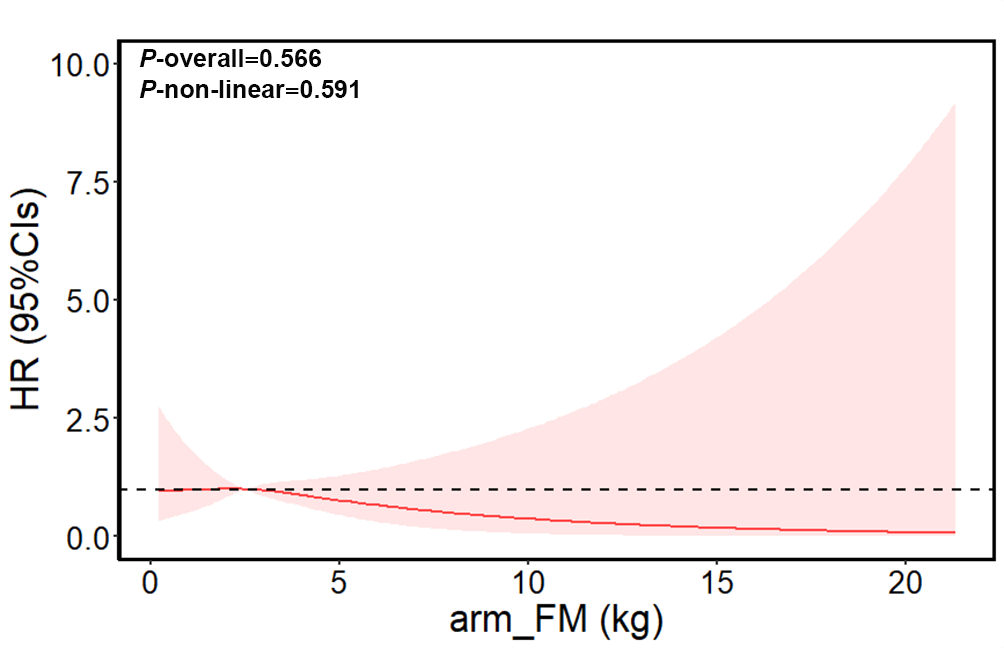

Supplement: Supplementary file 1 [file Data_Sheet_1.ZIP › Supplementary Figures/Supplementary Figure 3(A).tif]

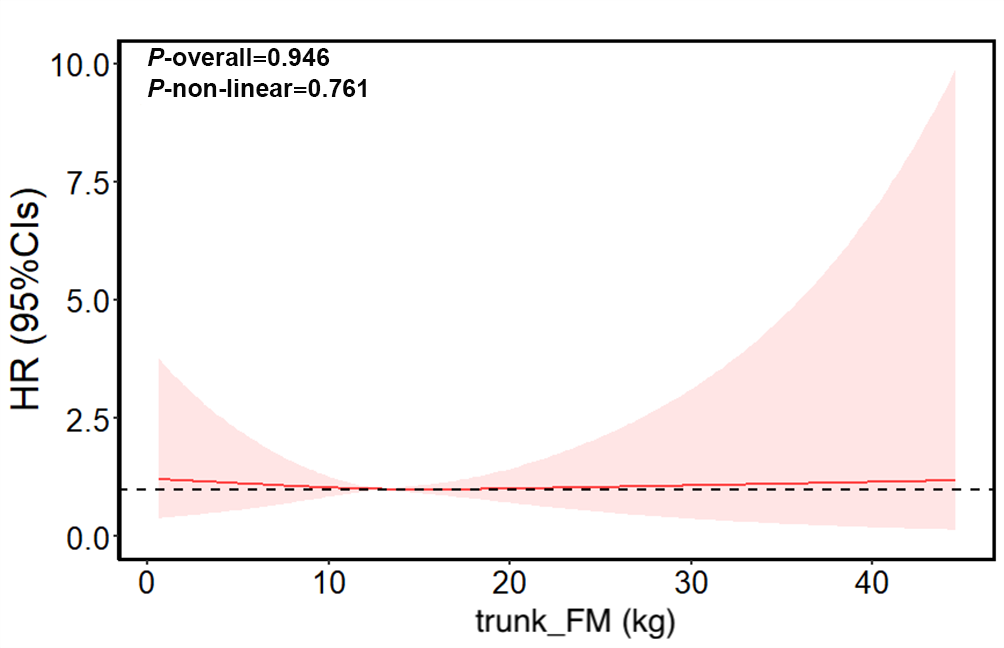

Supplement: Supplementary file 1 [file Data_Sheet_1.ZIP › Supplementary Figures/Supplementary Figure 3(B).tif]

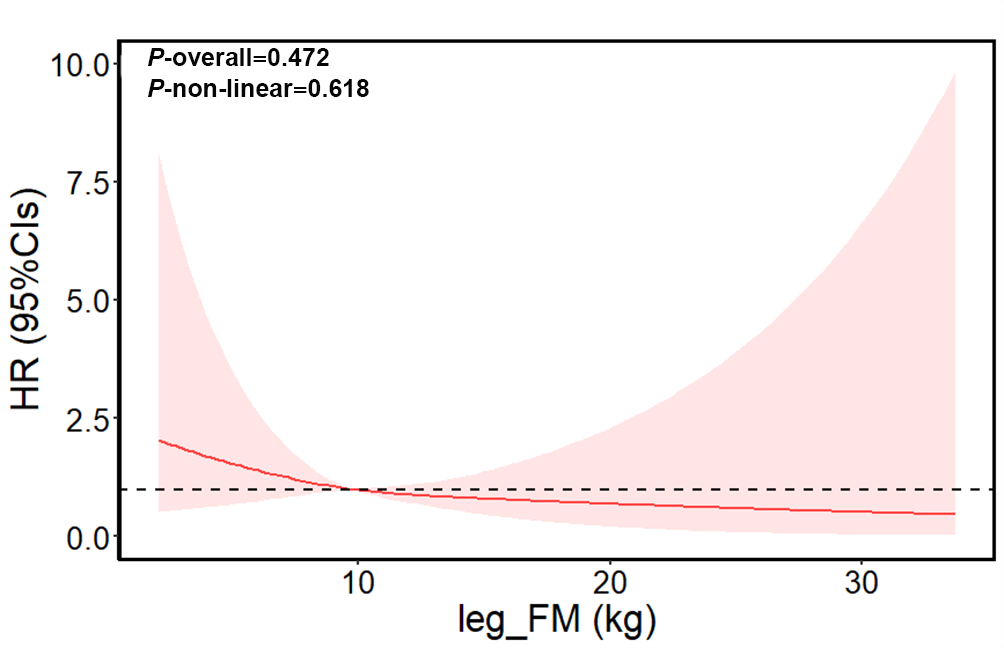

Supplement: Supplementary file 1 [file Data_Sheet_1.ZIP › Supplementary Figures/Supplementary Figure 3(C).tif]

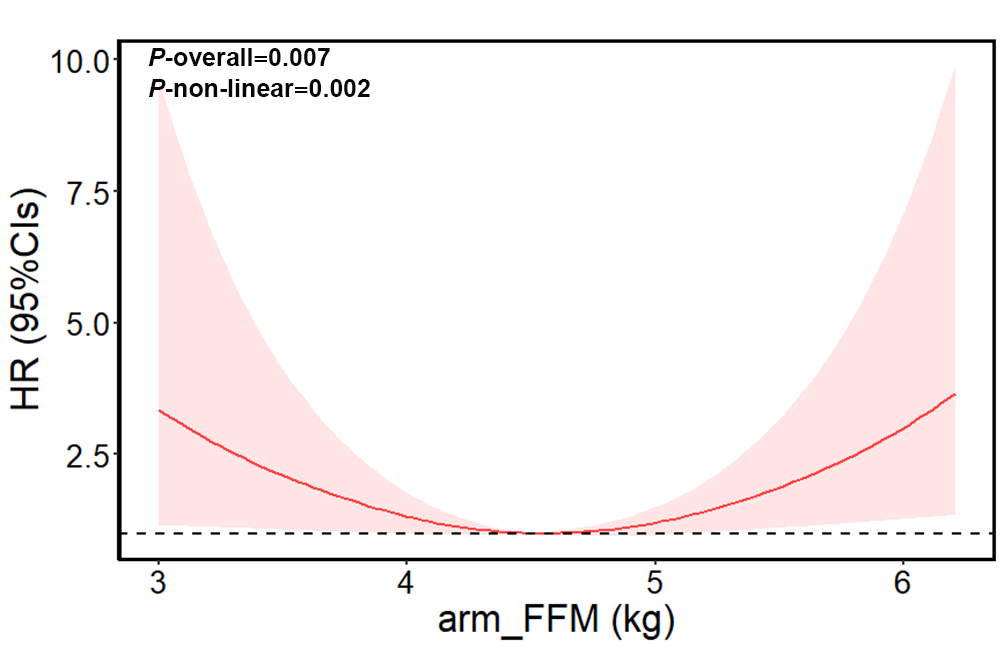

Supplement: Supplementary file 1 [file Data_Sheet_1.ZIP › Supplementary Figures/Supplementary Figure 3(D).tif]

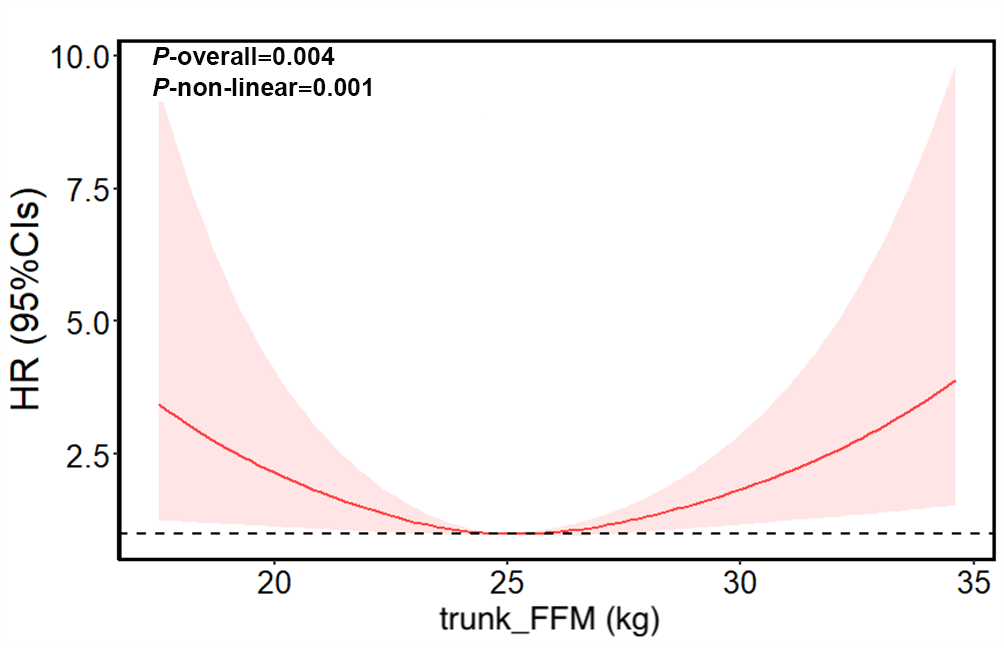

Supplement: Supplementary file 1 [file Data_Sheet_1.ZIP › Supplementary Figures/Supplementary Figure 3(E).tif]

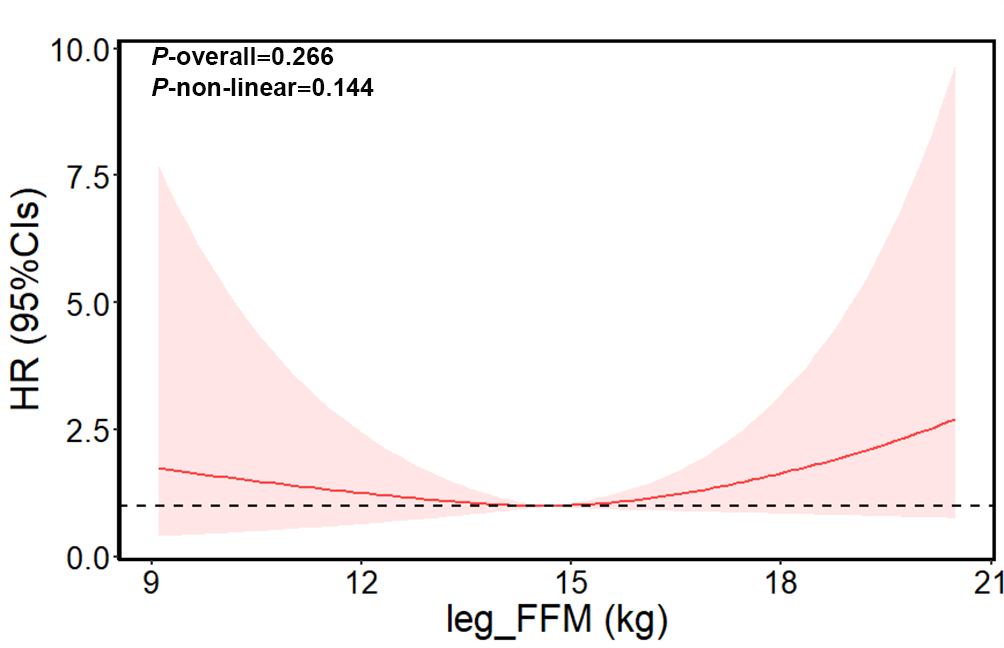

Supplement: Supplementary file 1 [file Data_Sheet_1.ZIP › Supplementary Figures/Supplementary Figure 3(F).tif]

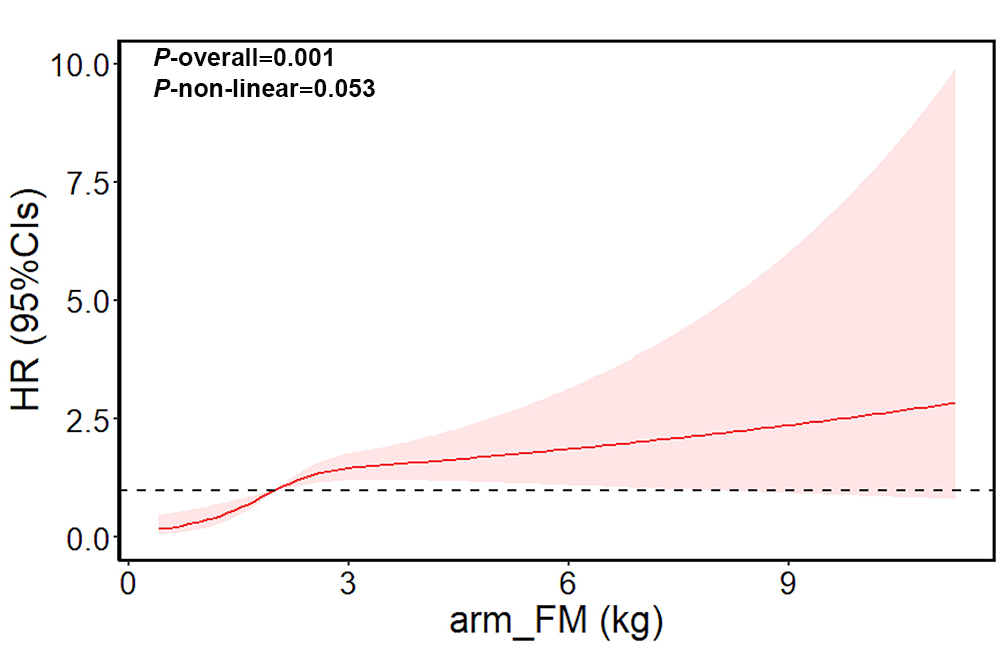

Supplement: Supplementary file 1 [file Data_Sheet_1.ZIP › Supplementary Figures/Supplementary Figure 4(A).tif]

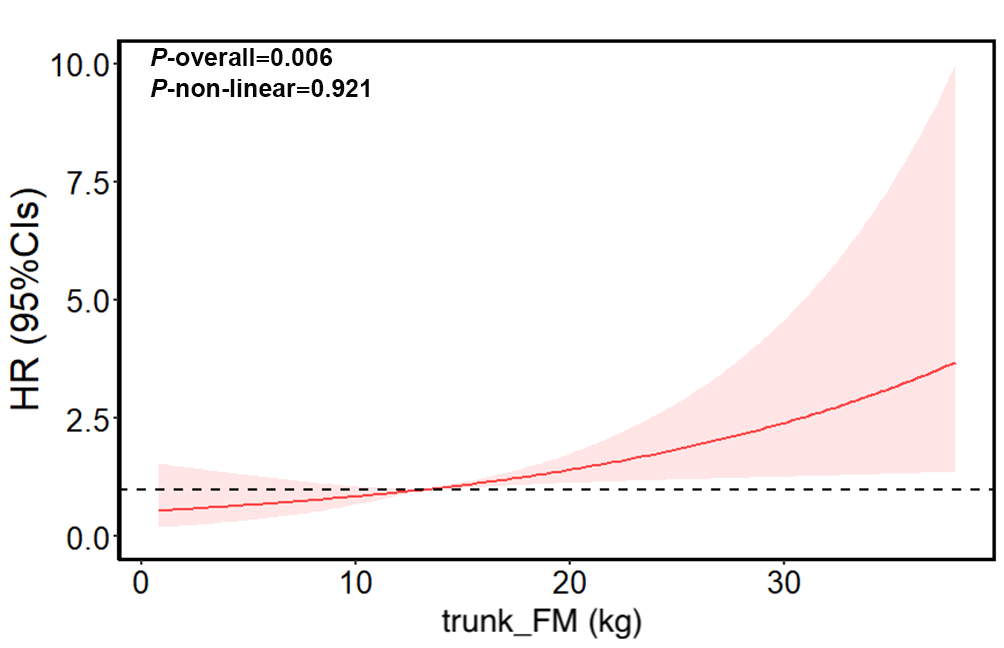

Supplement: Supplementary file 1 [file Data_Sheet_1.ZIP › Supplementary Figures/Supplementary Figure 4(B).tif]

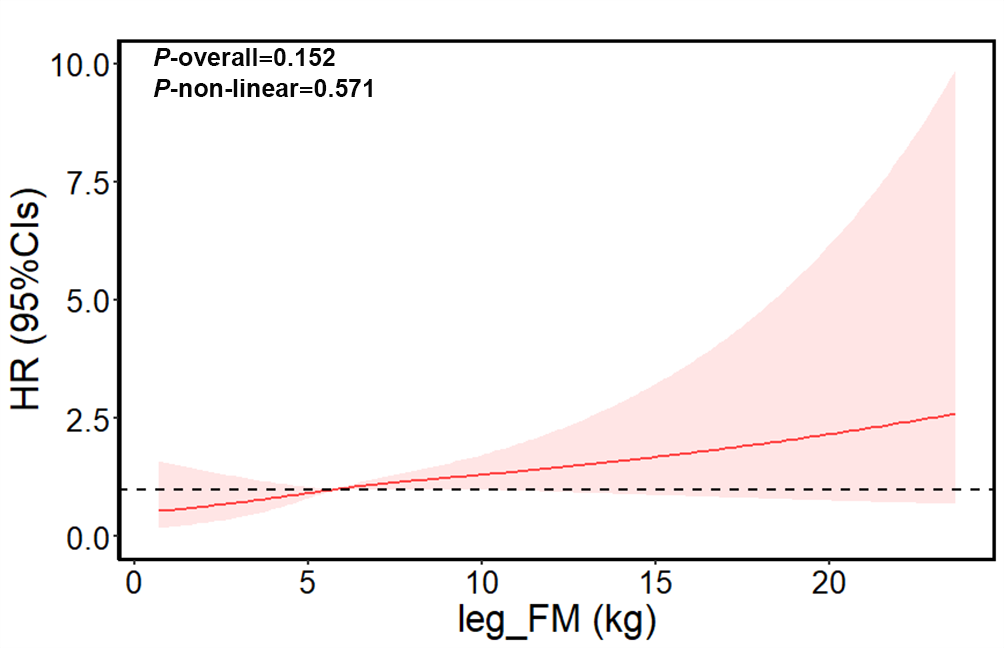

Supplement: Supplementary file 1 [file Data_Sheet_1.ZIP › Supplementary Figures/Supplementary Figure 4(C).tif]

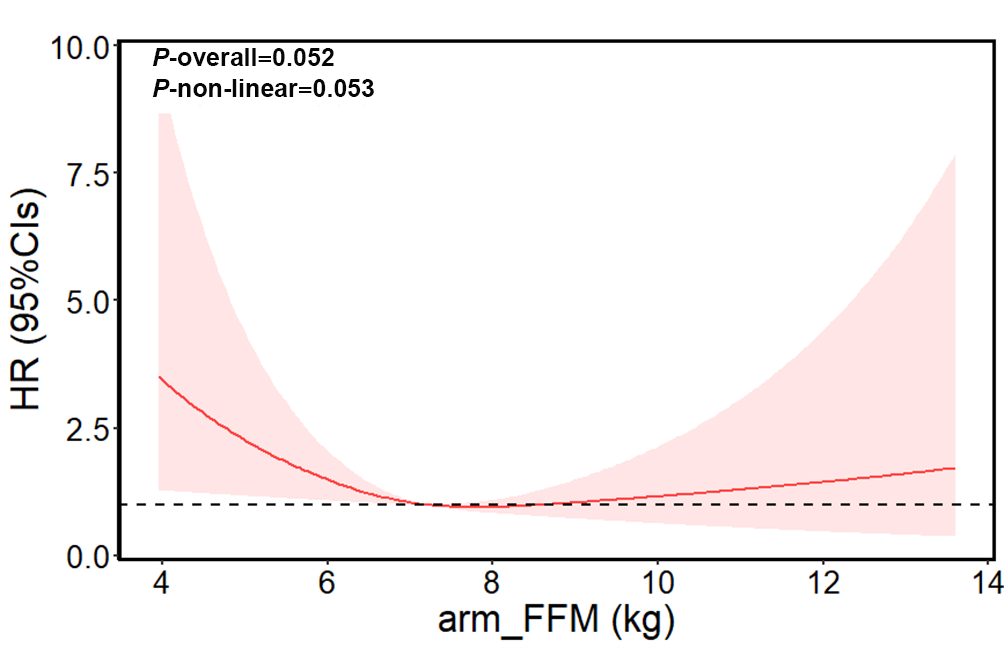

Supplement: Supplementary file 1 [file Data_Sheet_1.ZIP › Supplementary Figures/Supplementary Figure 4(D).tif]

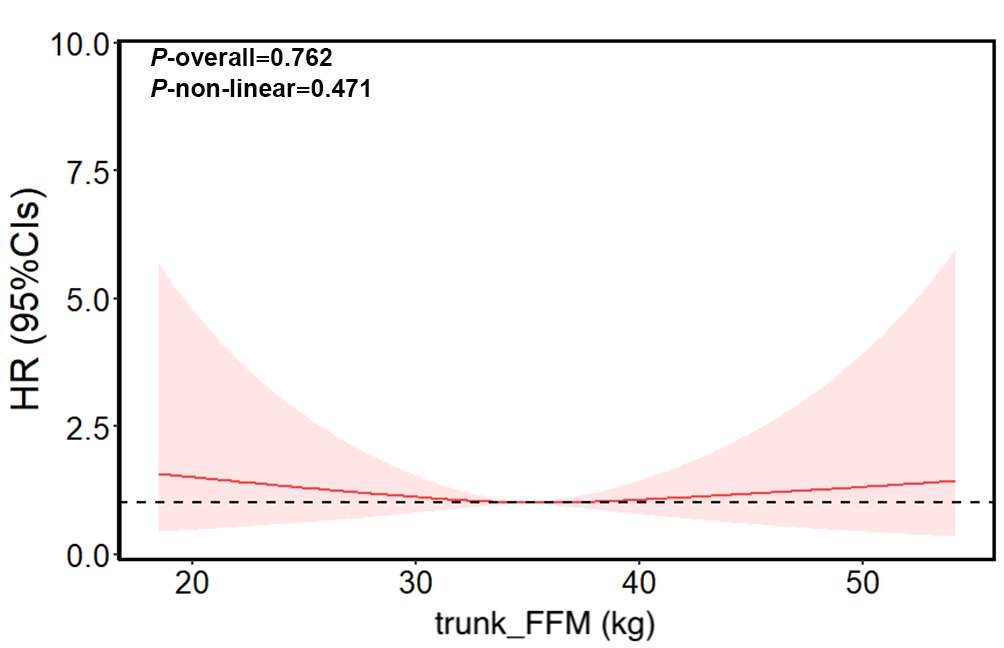

Supplement: Supplementary file 1 [file Data_Sheet_1.ZIP › Supplementary Figures/Supplementary Figure 4(E).tif]

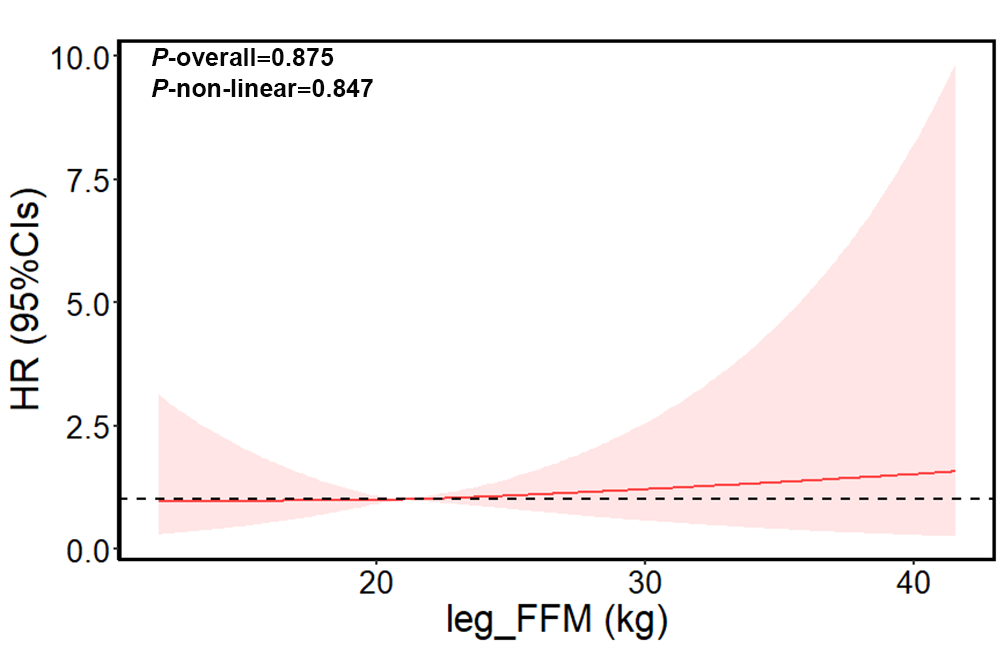

Supplement: Supplementary file 1 [file Data_Sheet_1.ZIP › Supplementary Figures/Supplementary Figure 4(F).tif]

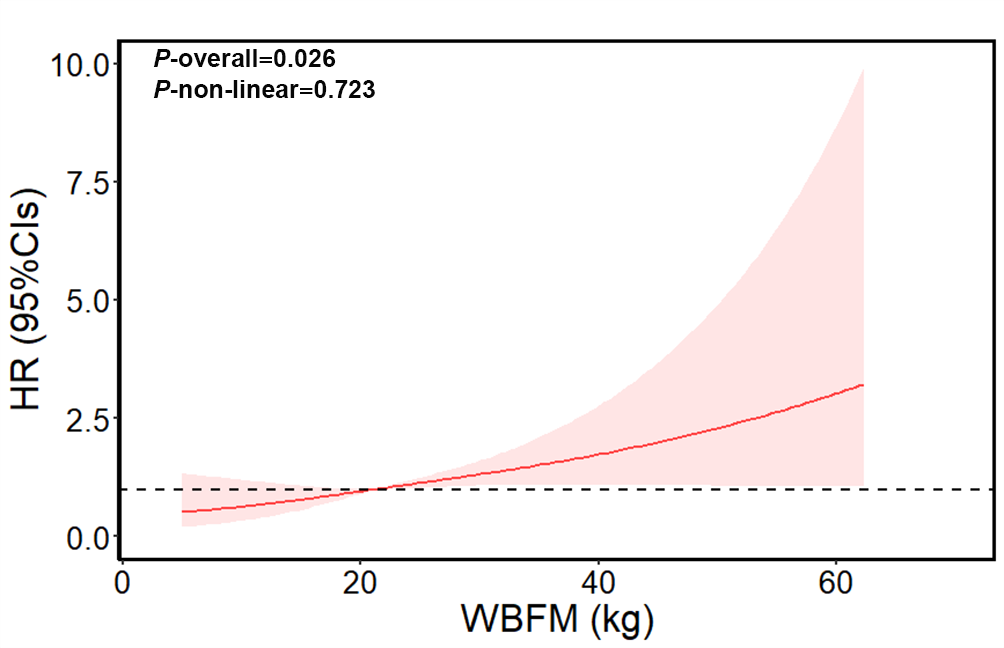

Supplement: Supplementary file 1 [file Data_Sheet_1.ZIP › Supplementary Figures/Supplementary Figure 5(A).tif]

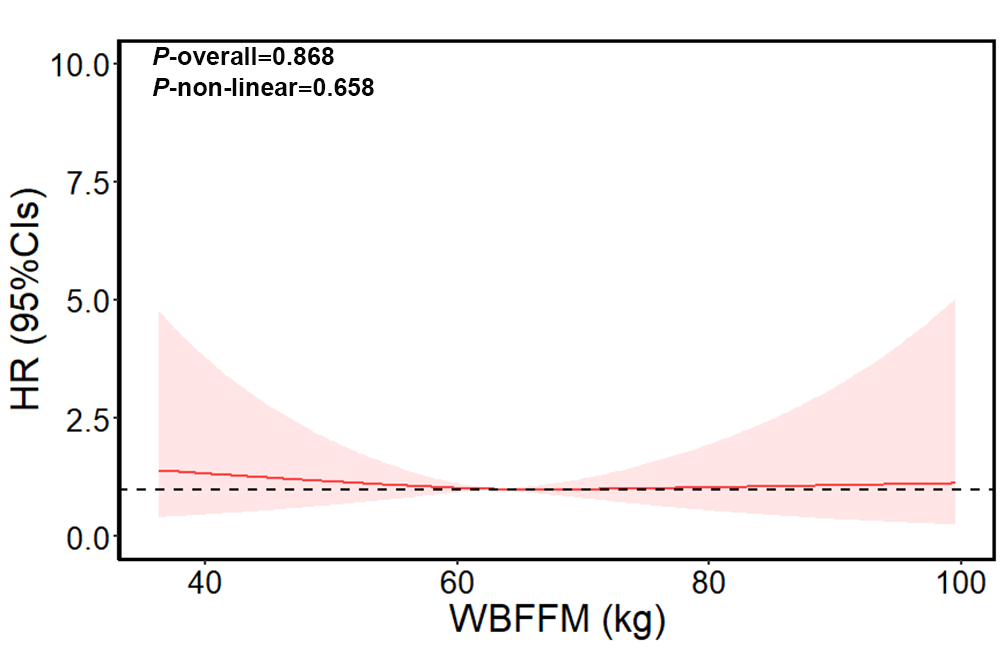

Supplement: Supplementary file 1 [file Data_Sheet_1.ZIP › Supplementary Figures/Supplementary Figure 5(B).tif]

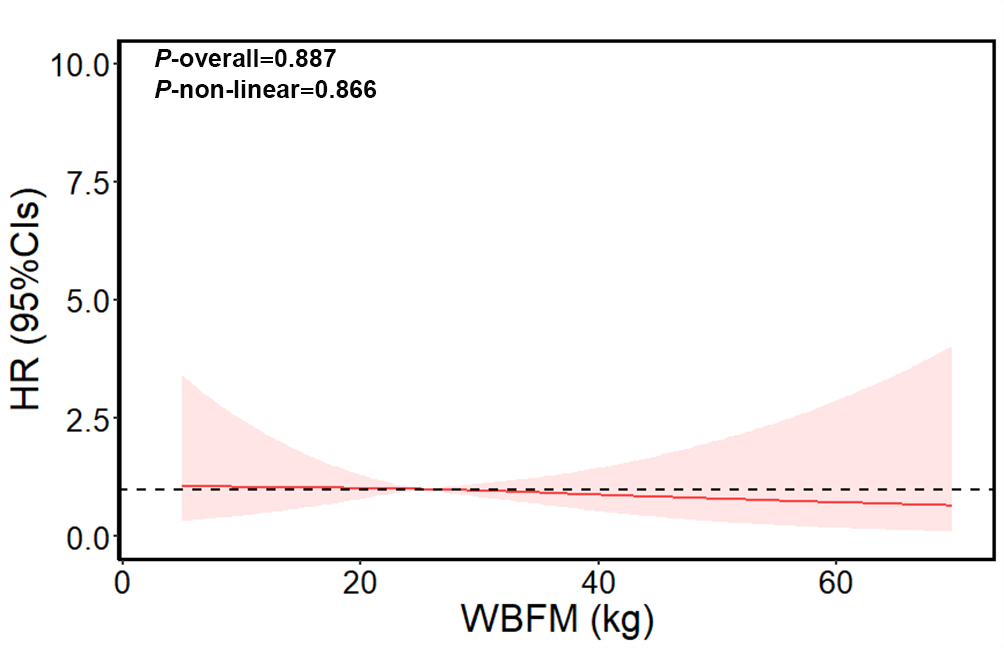

Supplement: Supplementary file 1 [file Data_Sheet_1.ZIP › Supplementary Figures/Supplementary Figure 5(C).tif]

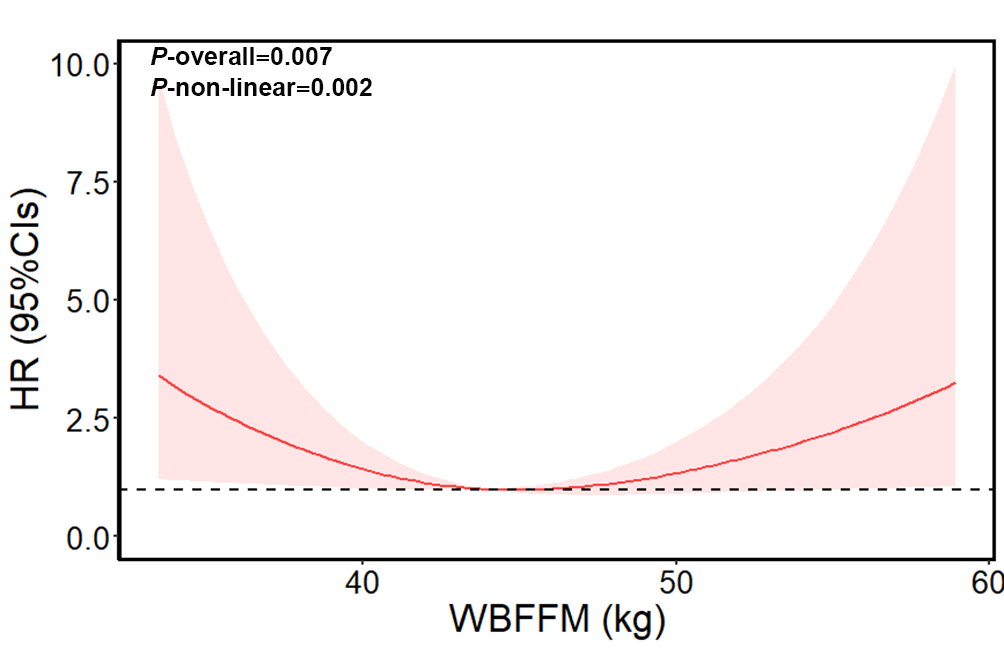

Supplement: Supplementary file 1 [file Data_Sheet_1.ZIP › Supplementary Figures/Supplementary Figure 5(D).tif]

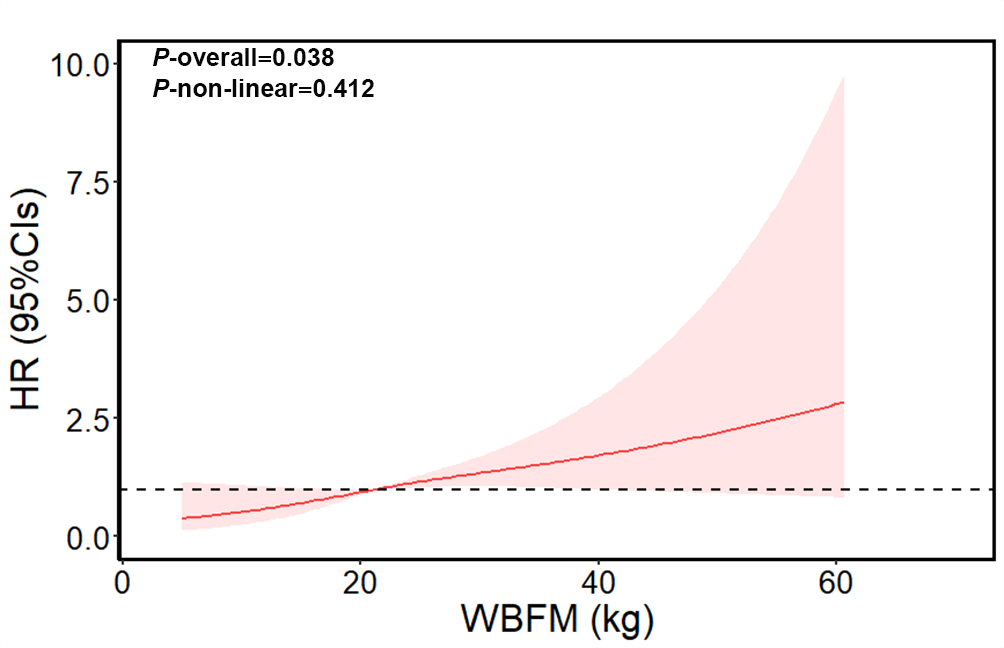

Supplement: Supplementary file 1 [file Data_Sheet_1.ZIP › Supplementary Figures/Supplementary Figure 5(E).tif]

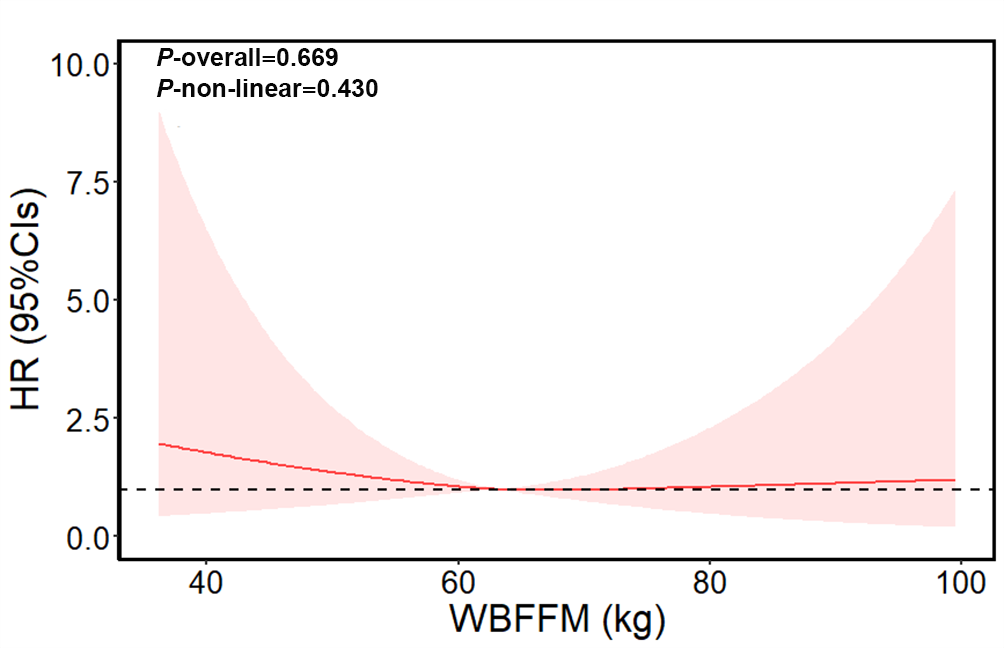

Supplement: Supplementary file 1 [file Data_Sheet_1.ZIP › Supplementary Figures/Supplementary Figure 5(F).tif]

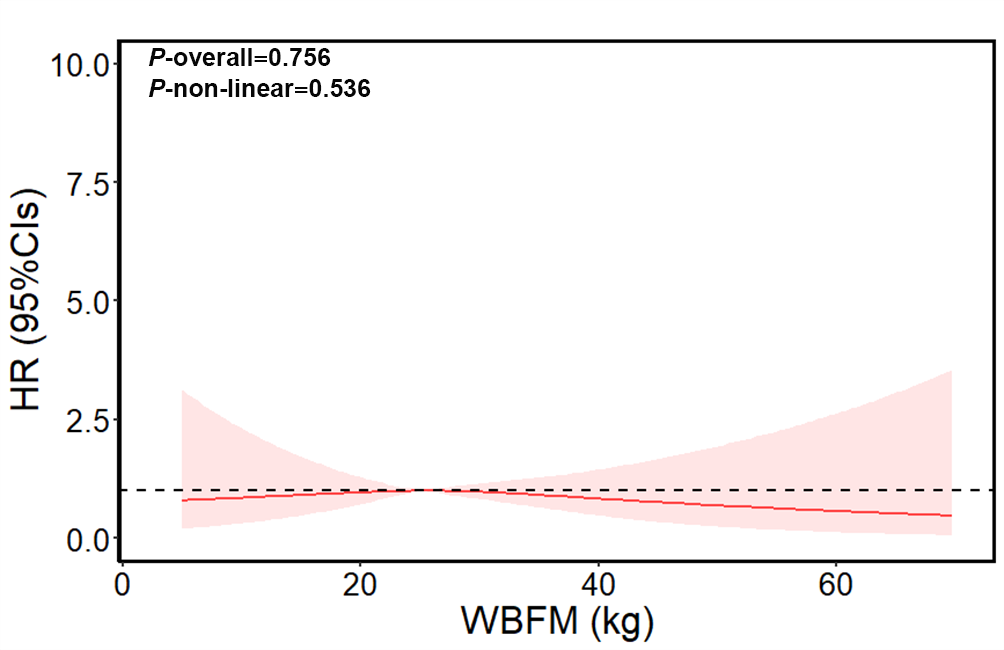

Supplement: Supplementary file 1 [file Data_Sheet_1.ZIP › Supplementary Figures/Supplementary Figure 5(G).tif]

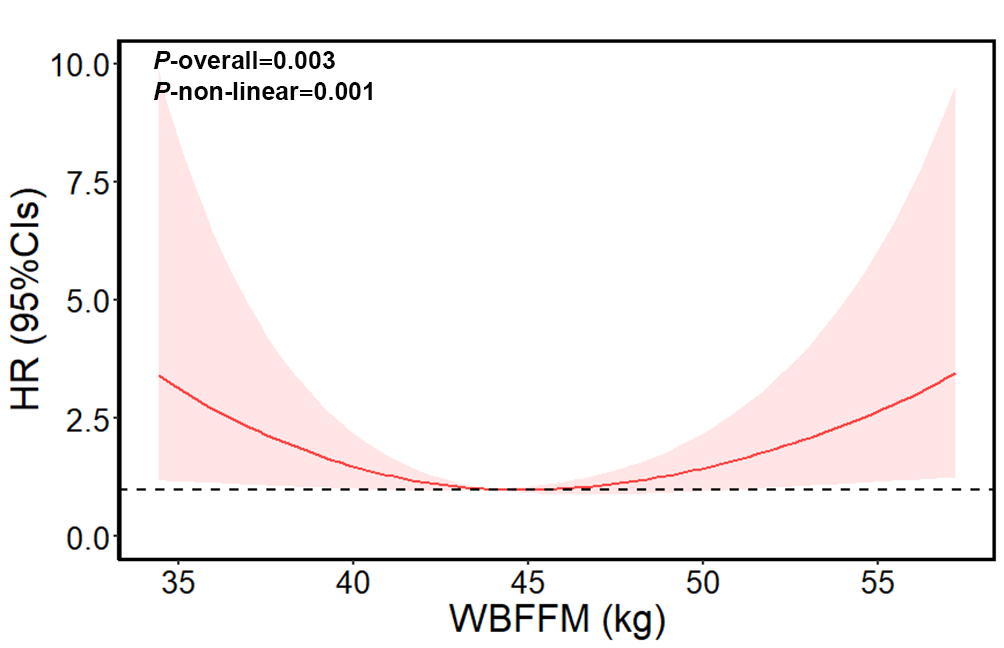

Supplement: Supplementary file 1 [file Data_Sheet_1.ZIP › Supplementary Figures/Supplementary Figure 5(H).tif]
